# Items

**Published:** 1846-03-01

**Authors:** 


*Items.*—The vacancy in the Transylvania Medical School, Lexington, Ky., has been filled by the election of Samuel Annan, M. D., of Baltimore. This selection was from forty-nine applicants, representing seventeen States.—Prof. Watson having resigned the office of Professor of Theory and Practice in the same Institution, Elisha Bartlett, M. D., has been elected to succeed him. Prof. B., who was formerly connected with this School, and lately with the Baltimore School, is now in Europe, but has signified his willingness to accept the place, and will return in season for the next session.—Drs. Goodyear and Hyde, of Cortlandville, have established a private School for the study of Anatomy and Surgery. We are glad to see this. Private medical schools are much needed. We hope it will be followed by others.—Dr. Knapp, of Chicago, Ill., has issued a prospectus for a new Medical Journal to be entitled the "North Western Journal of the Medical and Physical Sciences." It is to be a bi-monthly, containing 80 pages, octavo, at two dollars per annum.

